# Supplementary material for: Evaluation of a Physical Activity and Multi-Micronutrient Intervention on Cognitive and Academic Performance in South African Primary Schoolchildren
Source: Nutrients. 2022 Jun 23;14(13):2609. doi: 10.3390/nu14132609 (PMC9268611; doi:10.3390/nu14132609)
Supplement: Supplementary file 1 [file nutrients-14-02609-s001.zip › nutrients-1720773-supplementary.pdf]

Table S1. Intervention arms[1] at 4 primary schools in Nelson Mandela Bay, South Africa carried out between April 2019 and September 2019.

	Grade level			
**School**	**1**	**2**	**3**	**4**
**1**	PA + Placebo	PA + MMNS	MMNS	Placebo
**2**	Placebo	PA + Placebo	PA + MMNS	MMNS
**3**	MMNS	Placebo	PA + Placebo	PA + MMNS
**4**	PA + MMNS	MMNS	Placebo	PA + Placebo

Notes. [1] PA = Physical activity, MMNS = Multi-micronutrient supplementation, Placebo = Placebo control-group condition.

Table S2. Composition of the MMNS used in the intervention study.

Nutrient	Amount per tablet (mg)	Overage[1] %
**β-carotene (Provitamin A)**	3600 (equiv. to 2000 IU Vit. A)	60%
**Cholecalciferiol (Vit. D_3)**	0.010	60%
**Rac-α-tocopherylacetate, TE-3 (Vit. E)**	9.0	60%
**Thiamine (Vit. B_1)**	1.1	60%
**Riboflavin (Vit. B_2)**	0.5	60%
**Pyridoxine (Vit. B_6)**	0.5	60%
**Folic acid (Vit. B_9)**	0.2	60%
**Niacin (Vit. B_3)**	8.0	60%
**Vitamin B_{12}**	0.0012	60%
**Ascorbic acid (Vit. C)**	60	60%
**Iron (as NsFeEDTA)**	8.0	60%
**Iodite**	0.1	60%
**Selenium**	0.02	60%
**Zinc**	5.0	60%
**Carrier [2]**		

Notes. [1] Overage of 60% were added to ensure required amounts during 12-month shelf life. [2] Carrier is used to indicate that the amount of carrier needed to have a total of 1 kg/kg was added. Vit. = Vitamin, equiv. = equivalent, NsFeEDTA = sodium ferric ethylenediaminetetraacetate.
